# From Complex Behavior to New Drugs: Chemoinformatics Shows the Way

**DOI:** 10.1371/journal.pbio.1001713

**Published:** 2013-11-19

**Authors:** Richard Robinson

**Affiliations:** Freelance Science Writer, Sherborn, Massachusetts, United States of America

[Fig pbio-1001713-g001]If you are a medicinal chemist casting your net for new drugs, you have a choice: you can take a bottom-up approach, in which you begin with a molecule that hits a known molecular target and hope it translates eventually into a physiological effect; or you can take a top-down approach, in which you begin with a molecule that causes the desired physiological effect and then find the molecular target in order to better understand the effect and optimize your drug's interaction with it.

**Figure pbio-1001713-g001:**
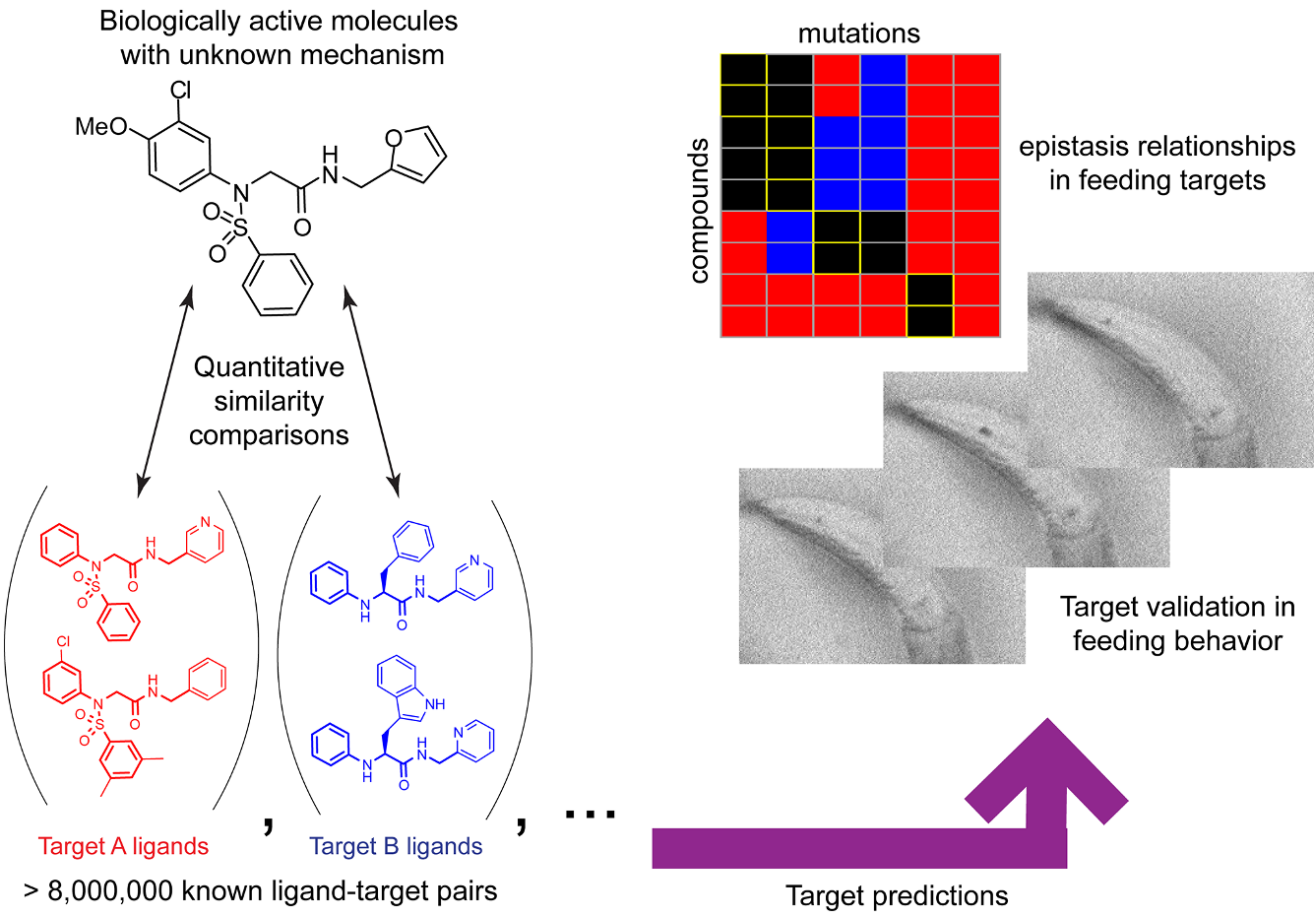
A computational approach to target identification is applied to elucidate the pharmacological targets underlying the regulation of feeding behavior in the nematode *C. elegans*.

This top-down approach, called a phenotypic screen, seems to be the most practical route when you want to alter a complex behavior, such as feeding, which involves multiple tissues and unknown molecular pathways. But after finding the right molecule, discovering the target it hits and the pathway it affects can be challenging. Additionally, these discovery steps are by necessity performed in a lab model, and the targets and pathways in the model must still be shown to be relevant to human physiology.

In a new study in this issue of *PLOS Biology*, George Lemieux, Bryan Roth, Brian Shoichet, Kaveh Ashrafi, and colleagues analyze a set of compounds emerging from a phenotypic screen of fat content in worms. By combining chemoinformatics, in vitro testing, and in vivo assays, they identify previously unknown pathways that affect feeding behavior in worms and that are likely to have important effects in mammals.

The authors began with a set of 84 compounds they had found altered fat metabolism in the worm. For all but one of these compounds, no biological targets were known. To find likely targets, they interrogated a database of almost 2,500 targets and more than 8 million ligand interactions with mostly mammalian targets, comparing the structure of their compounds with ligand sets in the database. They found possible targets for 79 of the 84 compounds, and chose 16 compounds for in vitro testing against the predicted mammalian targets, many of which were enzymes or membrane receptors.

Next, they returned to worms. In the original screen, fat accumulation (assayed by fluorescence microscopy) stood in for feeding. Here, they measured the rate of contraction of the pharynx—swallowing, essentially—for a more direct indication of the related physiology of feeding behavior. Four compounds stood out for their ability to increase feeding in larvae and adults.

But did these four do so through interacting with worm counterparts of mammalian targets that were identified? To find out, the authors knocked down the predicted target using RNA interference. They reasoned that if the compound interacted with the predicted target, its effect was almost certainly inhibitory (virtually all drugs inhibit, rather than facilitate, action of the biomolecules they interact with). Therefore, knocking down the predicted target should have the same effect as the compound, and furthermore, adding the compound shouldn't increase the effect, since the target would already be out of commission.

According to database predictions and in vitro testing, the compound D20 inhibited a human receptor tyrosine kinase (RTK) called Flt-3. Worms have no exact match, but they do have many similar RTKs. Testing each of them without and then with D20, they found that one, called ver-3, increased feeding when knocked down but was insensitive to additional D20, indicating that D20 acted on ver-3. Using the same strategy, the authors also showed that another compound, called F15, acted on a receptor similar to human oxytocin receptor, which is known to influence feeding behavior in mammals. Further analysis identified two other pathways, for a total of four, all previously undescribed with respect to feeding regulation in the worm and all with relevance to vertebrate feeding regulation. It is not yet clear whether the pathways affect feeding behavior in humans.

The strategy exploited in this study has wide applicability for discovering drugs for other behaviors, as long as a phenotypic correlate can be found and measured in an animal model. Not every compound identified will have a relevant human target, but if the success rate in future trials approximates that from this study, this method may prove to be a rich source of new compounds, targets, and pathways that can aid the design of the next generation of drugs for human use.

**Lemieux GA, Keiser MJ, Sassano MF, Laggner C, Mayer F, et al. (2013)** ***In silico*** **Molecular** **C** **omparisons of** ***C. elegans*** **and Mammalian Pharmacology Identify Distinct Targets that Regulate Feeding.** doi:10.1371/journal.pbio.1001712

